# Implementing a Functional Group Analysis Activity to Support Student Learning in Medicinal Chemistry: A Three-Year Experience

**DOI:** 10.3390/pharmacy13050126

**Published:** 2025-09-04

**Authors:** Ansel Belani, Jitendra D. Belani

**Affiliations:** Jefferson College of Pharmacy, Thomas Jefferson University, Philadelphia, PA 19107, USA; ansel.belani@jefferson.edu

**Keywords:** formative assessments, active learning, medicinal chemistry, peer-to-peer learning, student engagement

## Abstract

Many pharmacy students begin medicinal chemistry with limited experience in chemical structure interpretation and reactivity patterns, making it difficult to connect foundational concepts to real-world drug behavior. We introduced a low-stakes functional group analysis activity that included peer discussion and a follow-up quiz to improve learning and reduce student anxiety. We studied the impact of this activity by comparing Exam 1 scores across three cohorts: one before the activity (2022) and two after implementation (2023 and 2024). The average Exam 1 scores improved in the post-intervention years, and while the overall difference across cohorts did not reach statistical significance, post hoc analysis revealed a significant improvement between the 2022 and 2024 cohorts. The students who engaged more deeply, especially those who performed well on the quiz, consistently earned higher exam scores, with strong positive correlations observed in both years. These results suggest that simple, low-stakes activities that focus on core concepts can promote engagement and support student success, even in challenging, content-heavy courses like medicinal chemistry.

## 1. Introduction

Many pharmacy students feel that medicinal chemistry poses a significant challenge, particularly those entering the program with only minimal prior chemistry coursework. At our college of pharmacy, for example, applicants may be admitted with as little as a “C” in organic chemistry, which satisfies admissions criteria but often leaves them underprepared for the structure-heavy, theory-driven content of medicinal chemistry. As a result, concepts such as acid–base equilibria, pKa, or molecular interactions may seem abstract, fueling anxiety that interferes with learning and engagement. These challenges are compounded by the educational context: in the United States, pharmacy is a professional doctoral (PharmD) program typically entered after at least two years of undergraduate study, often with limited exposure to advanced chemistry. In contrast, in many countries such as the United Kingdom, pharmacy is an undergraduate qualification with higher entry thresholds in chemistry, and pass marks and grading schemes vary between institutions. Together, limited preparation, abstract conceptual content, and program structure help explain why students often find medicinal chemistry to be one of the more intimidating subjects in the curriculum.

Beyond these issues of preparation and confidence, students also struggle to interpret chemical structures in ways that connect directly to therapeutic reasoning. Many commonly prescribed drugs, such as levothyroxine, lisinopril, amoxicillin, tamsulosin, olmesartan, methotrexate, etc., contain multiple ionizable functional groups. These molecules often exist as zwitterions at physiological pH, and recognizing how their charge state shifts across environments is essential for predicting absorption, solubility, distribution, bioavailability, and renal excretion. Without explicit training in functional group analysis, students may not appreciate how chemical structure dictates clinical behavior, leaving them less equipped to reason through mechanisms of action or anticipate drug properties.

Medicinal chemistry is essential to the education of pharmacists, as it provides the foundation for understanding pharmacotherapy, drug action, and clinical decision-making [1,2]. At its core is functional group analysis, which determines how drugs behave in the body—shaping solubility, ionization, metabolism, absorption, and toxicity (ADMET)—as well as their interactions with molecular targets through non-covalent forces such as hydrogen bonding, dipole–dipole interactions, and van der Waals forces [3]. These structural features define pharmacokinetic and pharmacodynamic (PK/PD) profiles and are central to understanding drug action and structure–activity relationships (SARs) [4,5]. Students who lack a strong foundation in this area often struggle to make connections or reason through therapeutic mechanisms. Educational theory helps explain these struggles: constructivist perspectives emphasize the need to connect new ideas to prior knowledge, while cognitive load theory highlights how abstract content presented without scaffolding can overwhelm working memory [6,7]. Retrieval practice and peer learning have both been shown to mitigate these challenges [8,9], and a variety of pedagogical strategies have been explored, including case-based learning [10,11], problem-based laboratory activities [12], technology-enhanced collaboration [13], and gamified or virtual-reality platforms [14]. These interventions demonstrate the importance of structured, active learning opportunities that reduce anxiety while improving conceptual understanding. Our study builds on this work by introducing a low-stakes activity designed to integrate functional group analysis directly into the pharmacy curriculum.

In response to these challenges, a functional group analysis was introduced into the medicinal chemistry course, starting in 2023. The activity was designed to be an assignment of low stakes yet high engagement, allowing students to build their structure-based reasoning skills without fear of performing poorly [15]. Using principles from active learning and retrieval of newly learned information, the assignment encouraged students to become more interactive and take control of the content they studied [16,17]. Each student was assigned a unique drug molecule and asked to identify and analyze its key functional groups, predict the ionization and solubility of the drug under different pH conditions, and relate these properties to the molecule’s behavior in vivo. A demonstration video was provided for the students to ensure that the activity went smoothly, and students then posted their analyses to a discussion board on the Canvas learning management system [18].

Another important point to note was that the activity included collaborative peer-learning, in which students were expected to peer-review others’ analyses, engaging with various drug structures beyond their assigned molecules [19,20]. Instructional feedback was given to students throughout the activity, and students could revise their work based on their instructor’s and classmates’ advice. The next week, students completed an individual quiz in class that reinforced the same learned concepts but was on new structures to test their new capabilities.

This open-ended two-part design, a discussion-based analysis with the quiz students received a week later, created an environment that supported content mastery without the pressure of high-stakes grading. Low-stakes, practice-based learning has been shown to reduce anxiety, promote risk-taking, reduce fear of making mistakes, and improve conceptual retention in science education [21]. With medicinal chemistry, we found that this approach encouraged more effortful engagement with chemical principles and greater confidence in applying them.

This study aims to describe the implementation of this functional group analysis activity over three academic years (2022–2024) and evaluate its impact on student performance and engagement. Rather than simply testing a direct hypothesis, this study presents a case-based analysis, including information such as how the activity was implemented and administered, how it evolved, and what observable patterns and new insights were gained for teaching across those three years. We compare outcomes from a pre-intervention cohort from 2022 to two post-intervention cohorts from 2023 and 2024, to examine whether student participation in the activity correlates with an increase in Exam 1 performance. Through this study, we aim to explore whether a simple instructional strategy can support early success in a traditionally challenging area of the pharmacy curriculum.

## 2. Materials and Methods

The intervention consisted of two components: a discussion-based functional group analysis activity and a follow-up individual quiz, each contributing 2.5% to the overall course grade (5% total). In the U.S. context, these are often described as “formative” or “low-stakes” because their purpose is to provide practice and feedback with minimal impact on final grades. To avoid confusion, we clarify that both activities were graded but low-stakes, designed to encourage participation without creating high-performance pressure.

For the discussion activity, each student was assigned a unique drug molecule and tasked with completing an analysis of its structure in detail. Students were asked to identify functional groups, predict acid-base properties, assess hydrogen bonding capabilities, and interpret solubility behavior under varying pH conditions for their unique molecule. Students were also encouraged to use software tools such as LogD predictors to support their analyses. To allow students to understand how the activity was meant to be performed fully, a step-by-step instructional video was provided to guide students for their assignment, and this video remained the same between the 2023 and 2024 cohorts. A detailed version of the activity, including instructions, tool links, and an example is provided in the Supplementary Materials. The completed analyses were submitted to an online discussion board.

The drug molecules assigned to students were chosen to represent a broad range of therapeutic classes and structural features, including both ionizable and non-ionizable functional groups. Selection prioritized clinical relevance and familiarity, with many compounds drawn from drug classes students would encounter later in the semester and easily recognizable at the level of experience expected of P1 students in community or hospital pharmacy. Students received clear instructions and iterative feedback from the instructor, allowing them to revise their analyses until they were correct. This approach emphasized reasoning and clarity while normalizing mistakes as a critical part of learning, consistent with evidence that guided, feedback-rich practice supports long-term retention more effectively than minimal-guidance approaches [22]. To promote collaboration, students were also encouraged to review and comment on at least one classmate’s post. The instructor monitored these interactions and provided additional guidance as needed, enabling students to refine their work based on both peer and instructor feedback. While specific compounds varied slightly across cohorts due to differences in class size, the overall instructional structure and feedback process were maintained for both 2023 and 2024, aligning with the literature demonstrating that structured guidance fosters deeper conceptual understanding in chemistry education [19,23].

One week after the discussion, students completed a brief in-class quiz assessing their ability to apply their learned concepts to new drug structures. The quiz was held electronically and graded as a formative assessment; students who received 80% or higher received full credit, to display mastery of a concept without much penalization [24].

The primary outcome was the performance on Exam 1, which highly emphasized functional group recognition, acid-base behavior, and solubility/pKa-based reasoning. Exam 1 was administered to all three cohorts using the same format and core-learning objectives, with comparable question structures across all years. While each year’s cohort consisted of different students, the course was delivered by the same instructor, using the same learning objectives, assessment structure, and grading rubrics. This consistency supports comparability across cohorts, although individual differences in prior preparation, motivation, or baseline capabilities cannot be fully controlled for in this design. Grading rubrics and scoring procedures were also kept consistent throughout this time. The secondary outcome was each student’s performance on their quiz and discussion activity in the 2023 and 2024 cohorts. These scores were analyzed in correlation with Exam 1 outcomes to assess whether deeper engagement in a less stressful activity was associated with improved exam performance.

Descriptive statistics, such as the mean, standard deviation, and range of the collected data, were calculated for Exam 1 scores across all three cohorts. Independent-sample t-tests were used to compare the 2023 and 2024 cohorts to the 2022 baseline. Pearson correlation coefficients were used to evaluate the relationship between discussion and quiz scores for the cohorts where the activity was implemented. The scores for Exam 1 were compared between the pre-intervention cohort and the post-intervention cohorts. All analyses were completed using standard statistical software, and significance was assessed where *p* < 0.05.

No generative AI tools were used in the generation of data, figures, or statistical analysis. Grammarly was used only to assist with minor text editing and organization.

## 3. Results

### 3.1. Exam 1 Performance Across Cohorts

**Figure 1:**
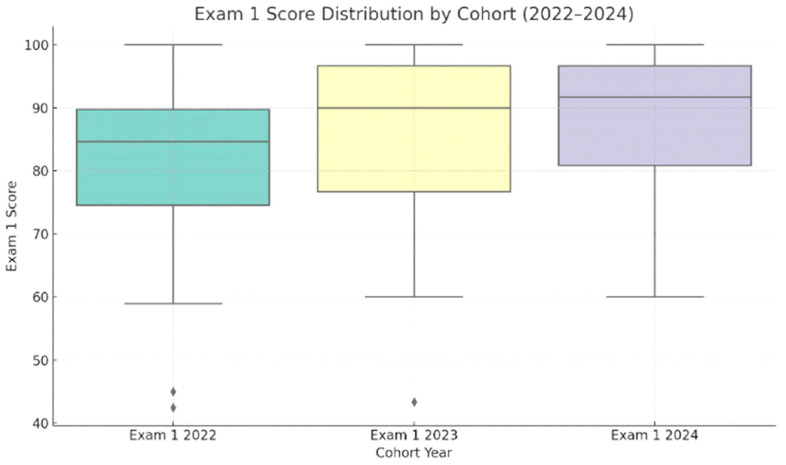
Distribution of Exam 1 scores across three cohorts (2022–2024)**.** Average scores increased following the implementation of the functional group analysis activity in 2023 and 2024, although differences did not reach statistical significance. Boxplots display the median, interquartile range, and outliers, with each point representing an individual student score.

Mean Exam 1 scores were compared across three cohorts: the spring semester section of 2022 was a pre-intervention sample, while the spring semester sections of 2023 and 2024 were post-intervention samples. The 2022 cohort had a mean score of 81.16 on Exam 1, where the standard deviation was 14.10, while the 2023 and 2024 cohorts had mean scores of 85.48 and 88.04, with standard deviations of 14.87 and 10.31, respectively (Table 1). A one-way ANOVA was conducted to compare Exam 1 scores across three cohorts of students (2022, 2023, and 2024) to determine whether the implementation of a functional group analysis activity had a measurable impact. Although there was a noticeable upward trend in mean scores, from 81.17 in 2022 to 85.48 in 2023 and 88.04 in 2024, the difference across cohorts approached but did not reach statistical significance; F(2, 112) = 2.95 and *p* = 0.056 (Figure 1).

Post hoc comparisons using Tukey’s HSD test revealed a statistically significant difference between the 2022 and 2024 cohorts (Q = 3.42; *p* = 0.045), with students in 2024 outperforming those in 2022 following the introduction of the activity. No significant differences were observed between 2022 and 2023 (Q = 1.95; *p* = 0.36) or between 2023 and 2024 (Q = 1.20; *p* = 0.66). Overall, these results suggest that cohort-level improvements were modest, and the overall ANOVA did not reach statistical significance. However, the significant gain between 2022 and 2024 points to a positive trend that may reflect growing student familiarity with structured active learning strategies or refinement of the activity’s implementation over time. This pattern suggests that the intervention contributed to a meaningful improvement, particularly between the first and third year of its use.

### 3.2. Engagement and Performance Within Post-Intervention Cohorts

**Figure 2:**
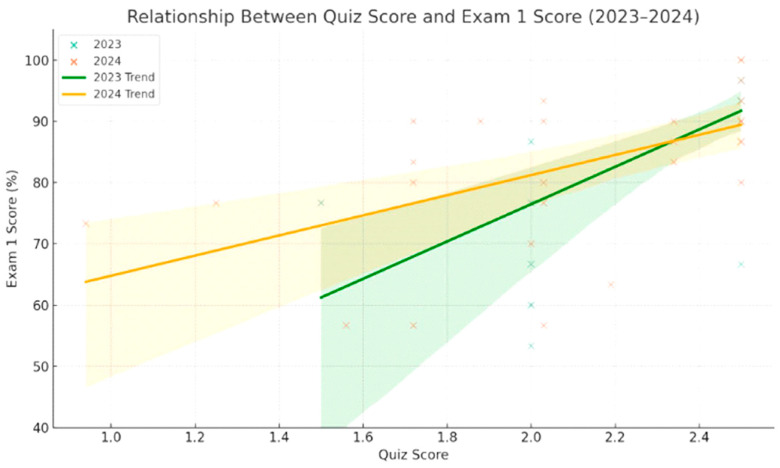
Relationship between quiz performance and Exam 1 scores in 2023 and 2024. A positive linear association was observed in both years, with stronger performance on the functional group quiz predicting higher exam scores. Regression lines are shown by cohort to illustrate trend differences.

To determine whether student participation in the functional group analysis activity was associated with a stronger exam performance, individual scores from the activity’s two components, the discussion board and quiz, were analyzed in relation to Exam 1 scores in the 2023 and 2024 cohorts. In both years, a moderate to strong positive correlation was observed between the quiz performance and Exam 1 scores (Figure 2).

- 2023: r = 0.67; *p* < 0.0001.
- 2024: r = 0.55; *p* = 0.0016.

These results suggest that students who performed well on the formative quiz, which emphasized the application of functional group concepts, were more likely to succeed on the summative exam, Exam 1. A weaker but still meaningful correlation was also observed between discussion board participation and Exam 1 scores.

- 2023: r = 0.43; *p* = 0.016.
- 2024: r = 0.40; *p* = 0.027.

Engagement with the activity was high: in 2023, 31 students generated 65 discussion board entries, and in 2024, 46 students generated 129 entries. These entries included initial submissions, instructor feedback exchanges, and subsequent revisions, with all students completing their required analyses and many refining their work in response to feedback. While students were encouraged to provide peer-to-peer comments, this was not required, and participation in that element was limited; therefore, peer commenting was not included in our statistical analysis. Even so, the results suggest that low-stakes assignments with open-ended, high-engagement tasks can meaningfully reinforce learning, particularly when paired with short formative quizzes. And finally, the in-class quizzes consisted of 20 multiple-choice questions to be completed within 50 min of a regular lecture time. Each item was designed to assess recognition and application of functional group properties, and new drug structures were used on each quiz to prevent memorization of examples and strengthen conceptual mastery by requiring transfer of knowledge to novel contexts [25]. Analysis of score distributions suggested that the activity was particularly beneficial for students in the middle performance range. High-achieving students continued to perform well regardless of participation, while lower-performing students showed noticeable improvements. These findings indicate that the activity may help reduce performance gaps by providing structured opportunities for practice that might not otherwise occur [21].

### 3.3. Summary

While cohort-level improvements in Exam 1 scores were not the most statistically significant, performance gains in the post-intervention years (2023 and 2024) were still consistent and accompanied by strong individual associations between engagement, measured through quiz performance, and assessment scores. These results suggest that the activity may have improved conceptual understanding, even without a formal grading or high-stakes incentive.

## 4. Discussion

The addition of a functional group analysis activity in a required medicinal chemistry course for a Doctor of Pharmacy program aimed to address an ongoing challenge in pharmacy education: engaging students with limited chemistry backgrounds in a subject that relies heavily on structural reasoning. While quantitative comparisons across the three cohorts did not yield statistically significant differences in average exam scores, the consistent upward trend in performance and the strong correlations between engagement levels and exam outcomes suggest that the functional group analysis activity had a meaningful educational impact. The overall differences across cohorts were modest, with statistical significance observed only between 2022 and 2024. Several factors may explain this pattern. First, relatively small sample sizes limited statistical power. Second, variability in baseline preparation may have contributed to year-to-year differences independent of the intervention. Finally, the benefits of the activity may have become more pronounced after multiple years of refinement and student familiarity with active learning methods.

One of the most convincing findings was the strong individual-level association between quiz performance and Exam 1 scores. This reinforces what many educators recognize: when students are given opportunities to engage with material in a low-stakes, structured format and then asked to apply it shortly after, their understanding deepens. The quiz did not introduce new content but asked students to transfer knowledge from the functional group analysis activity to new molecules. That such a simple exercise correlated with higher exam scores underscores the value of formative assessment and retrieval practice for reinforcing complex concepts like acid–base behavior or drug solubility.

The discussion component of the activity also showed a positive correlation with exam performance. Using an asynchronous discussion board motivated students to articulate their reasoning, critique peers’ work, and refine their own ideas. Although many students hesitated to post publicly, the low-stakes nature of the task and the opportunity to revise after feedback appeared to lower anxiety and encourage participation. This peer-review element broadened exposure to a wider range of drug structures and normalized revision and struggle as integral parts of learning.

From the instructors’ perspective, the activity is both scalable and sustainable. In cohorts of 31–46 students, individualized feedback was feasible, but larger groups may require additional support. Structured grading rubrics, guided peer feedback, or automated tools within the learning management system could help maintain meaningful feedback while reducing workload. Beyond feasibility, the activity requires minimal instructor time after initial setup, aligns closely with course objectives, and encourages independent as well as collaborative thinking. Most importantly, it fosters higher-order reasoning in an environment where students feel comfortable exploring ideas without the pressure of grades.

When viewed in the broader context of the pharmacy education literature, the functional group activity appears to offer an appealing balance between rigor and feasibility. Other strategies, such as virtual reality, team-based case studies, or gamified quizzes, have shown promise but often require substantial institutional investment or instructor preparation [14]. By contrast, our discussion-plus-quiz model can be implemented with minimal resources while still leveraging principles of retrieval practice and peer-to-peer learning. Anecdotally, students expressed appreciation for the chance to “make mistakes without penalty,” noting in informal conversations and emails that the exercise reduced their anxiety going into the first exam.

Several limitations should be noted. This study was conducted at a single institution in a course taught by the same instructor, limiting generalizability. Year-to-year comparisons are also confounded by differences in student preparation and motivation, even with consistency in instruction and assessments. Exam 1, while aligned with the activity, was the only formal measure of learning and may not have captured long-term retention. No qualitative data were collected through surveys or interviews, which could have provided deeper insight into how the activity shaped students’ confidence, anxiety, or perceptions of medicinal chemistry. In addition, while nearly all students completed and revised their analyses, participation in the online discussion board was limited. Anecdotal evidence suggested that students often collaborated offline, but this was not systematically captured. Future iterations could incorporate structured peer-commenting requirements and explicit tracking of both online and offline collaboration [26].

Future work could also include pre-/post-tests within a single cohort, longitudinal tracking, or rubrics for peer feedback to provide more robust within-subject evidence. Collecting direct student feedback would add valuable perspectives and complement quantitative data. Finally, adapting this activity beyond functional group analysis—for example, to drug–receptor interactions, metabolism, or therapeutic class comparisons—could extend its impact. The structured discussion-plus-quiz format appears well suited to reinforcing conceptual reasoning while maintaining the benefits of low-stakes, structured engagement.

## 5. Conclusions

This three-year case study explored the addition of a low-stakes, functional group analysis activity in a required medicinal chemistry course for a Doctor of Pharmacy program. Although average exam scores across cohorts increased only slightly, the consistent trend toward higher performance and the stronger individual-level associations between engagement in the activity and exam outcomes suggest that the activity genuinely supported student learning.

By combining guided individual analysis of molecule structure, peer discussion and review by classmates and the instructor, and a follow-up quiz, the activity allowed students to build confidence with foundational concepts effectively. Also importantly, the activity structure and its implementation in the course proved to be scalable, adaptable, and feasible for students with an already demanding course schedule.

For instructors facing the same problem or similar challenges, such as limited student knowledge in chemistry or students struggling with structural reasoning concepts, this activity offers a practical approach that reinforces conceptual understanding while lowering the pressure on students to do well. When implemented consistently and potentially paired with additional measures to ensure material retention, it has the potential to deliver deeper, long-term learning gains. As pharmacy curricula continue to emphasize the integration of foundational sciences with clinical application [27], scalable approaches like this can play an important role in bridging the gap and preparing students for more informed engagement in pharmacotherapy.

## Figures and Tables

**Table 1:** Summary of Exam 1 performance by cohort (2022–2024).

Cohort	Mean Exam 1	Standard Deviation	*n*
2022	81.16	14.10	38
2023	85.48	14.87	31
2024	88.04	10.31	46

## Data Availability

The data presented in this study is available on request from the corresponding author. The data is not publicly available due to institutional restrictions and the need to protect student privacy in accordance with IRB exemption guidelines.

## Supplementary Materials

The following supporting information can be downloaded at https://www.mdpi.com/article/10.3390/pharmacy13050126/s1. File S1: Full description of the functional group analysis activity.
