# Supplementary material for: Implementing a Functional Group Analysis Activity to Support Student Learning in Medicinal Chemistry: A Three-Year Experience
Source: Pharmacy (Basel). 2025 Sep 4;13(5):126. doi: 10.3390/pharmacy13050126 (PMC12452344; doi:10.3390/pharmacy13050126)
Supplement: Supplementary file 1 [file pharmacy-13-00126-s001.zip › pharmacy-3831612-supplementary.pdf]

Published Edit

Functional Group Analysis

All Sections

Jan 13 at 1:22am

114

The due date for completing the assignment is January 26th, 11:59 pm, 2024.

You are assigned a drug molecule (Please see the attached file). Please complete the following activities for that particular drug molecule:

1. Search the web for its structure and attach it to your answer.
2. Name Acidic Functional Groups present, identify as hydrogen bond donor/acceptor/neither
3. Name Basic Functional groups present, identify as hydrogen bond donor/acceptor/neither
4. Name Neutral Functional Groups present, identify as hydrogen bond donor/acceptor/neither
5. Name the heterocycles present in the molecule (aromatic and non-aromatic heterocycles from the select covered in class)
6. Does the molecule have chiral centers? Provide the total number of chiral carbons in the molecule and label **at least one** chiral center in the structure as R or S. In case the drug is available as a racemic mixture, please state that it is a racemic mixture.
7. Can the molecule exist as a zwitterion?
8. Create and activate an account on Chemaxon.com. Visit: https://account.chemaxon.com/register (https://account.chemaxon.com/register)
9. Using a LogD predictor (https://disco.chemaxon.com/calculators/demo/plugins/logd/ (https://disco.chemaxon.com/calculators/demo/plugins/logd/)), obtain the relationship between LogD and pH. Describe in brief the ionization of the drug molecule, and comment on pH where it is most lipophilic and the most hydrophilic.
10. Use the solubility predictor (https://disco.chemaxon.com/calculators/demo/plugins/solubility/ (https://disco.chemaxon.com/calculators/demo/plugins/solubility/)) and obtain the relationship between solubility and pH. Describe in brief how solubility changes with pH.

Websites you may Need for Q8 and Q9:

1. http://www.cheminfo.org/flavor/cheminformatics/Molfile/Generate_molfiles/index.html (http://www.cheminfo.org/flavor/cheminformatics/Molfile/Generate_molfiles/index.html)

2. https://pubchem.ncbi.nlm.nih.gov/ (https://pubchem.ncbi.nlm.nih.gov/)

Step-By-Step Demonstration:

https://jefferson.hosted.panopto.com/Panopto/Pages/Viewer.aspx?id=59dcf9f7-4100-406c-acdb-af9301100f9b (https://jefferson.hosted.panopto.com/Panopto/Pages/Viewer.aspx?

id=59dcf9f7-4100-406c-acdb-af9301100f9b)

Individual Assignments:

Functional Group Assignment-2.pdf
(https://jefferson.instructure.com/courses/38086/files/10794416?wrap=1)

Search entries or author | Unread | Subscribed

Reply

https:// Jitendra Belani (https://jefferson.instructure.com/courses/38086/users/233)

Jan 13, 2024

1. **Cephalexin:**

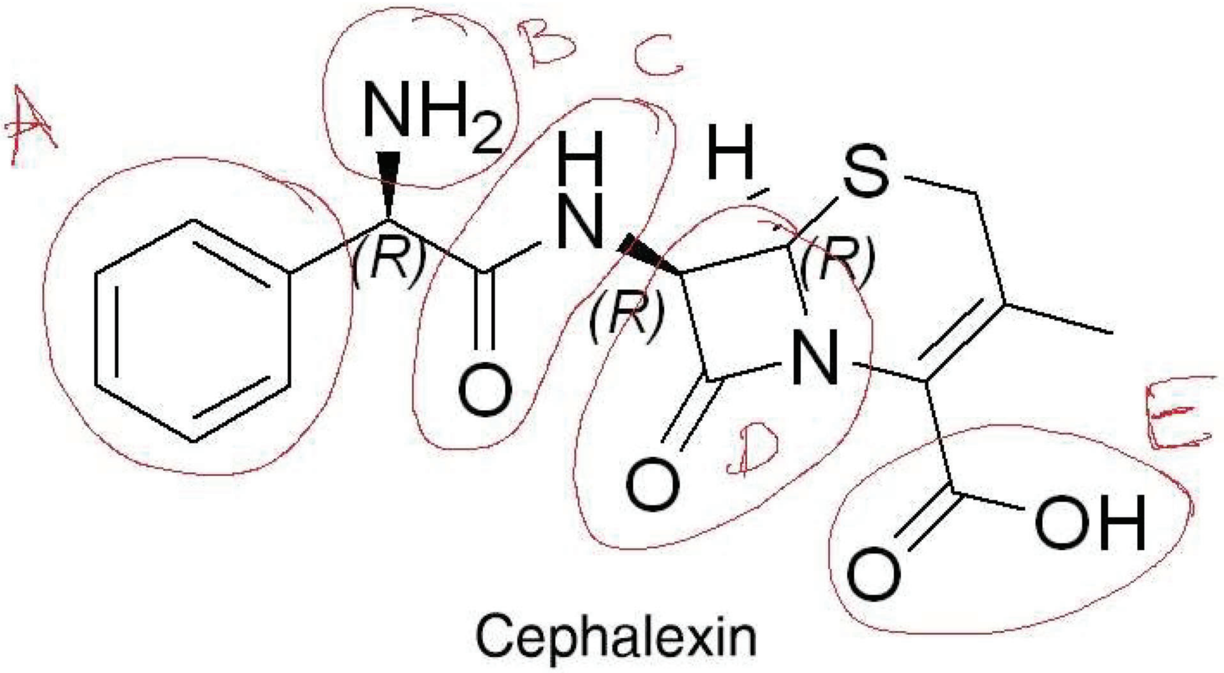

Cephalexin

2. **Acidic Functional Groups**: a) Carboxylic acid (**E**) , hydrogen bond donor and acceptor
3. **Basic Functional Groups**: a) Primary amine (**B**), hydrogen bond donor and acceptor
4. **Neutral Functional Groups**: a) Secondary amide (**C**), hydrogen bond donor and acceptor; b) Beta Lactam (**D**), hydrogen bond acceptor
5. **Heterocycles**: beta-lactam ring, dihydro-thiazine ring
6. Cephalexin has three chiral centers. In this case, all chiral centers have R configuration and are labeled in the structure above.

7. **Yes,** the molecule can exist as a zwitterion. It has one acidic functional group and one basic functional group.
8. **LogD and pH**: Lower the logD value more soluble the drug is in a buffer at that pH. Higher the logD value, more permeable the species is at that pH. The compound is *zwitterion*; contains both a basic and an acidic group, the isoelectric point of cephalexin in water is approximately 4.5 to 5. At acidic pH, the amine functional group will be ionized. At basic pH, the carboxylic acid will be ionized. The compound is predicted to have good solubility in all pH values, however it has the highest solubility in basic pH. The drug will be least soluble, most lipophilic and most permeable at its isoelectric point, around pH = 5 where the molecule exists as a zwitterion.

9. 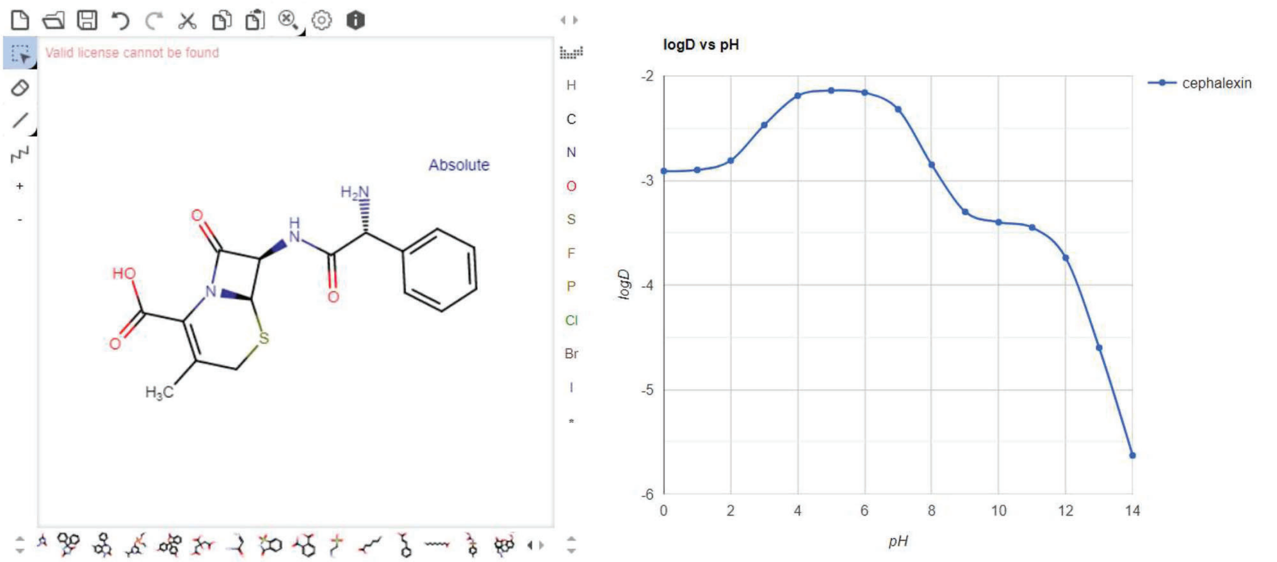

10. **LogS vs pH**: Since the molecule is a zwitterion, it is least soluble around the isoelectric point of about 5. Highest solubility is seen at pH > 11. Solubility also increases below pH of 4 where the amine is ionized. Solubility increases as the carboxylic acid group is completely ionized.

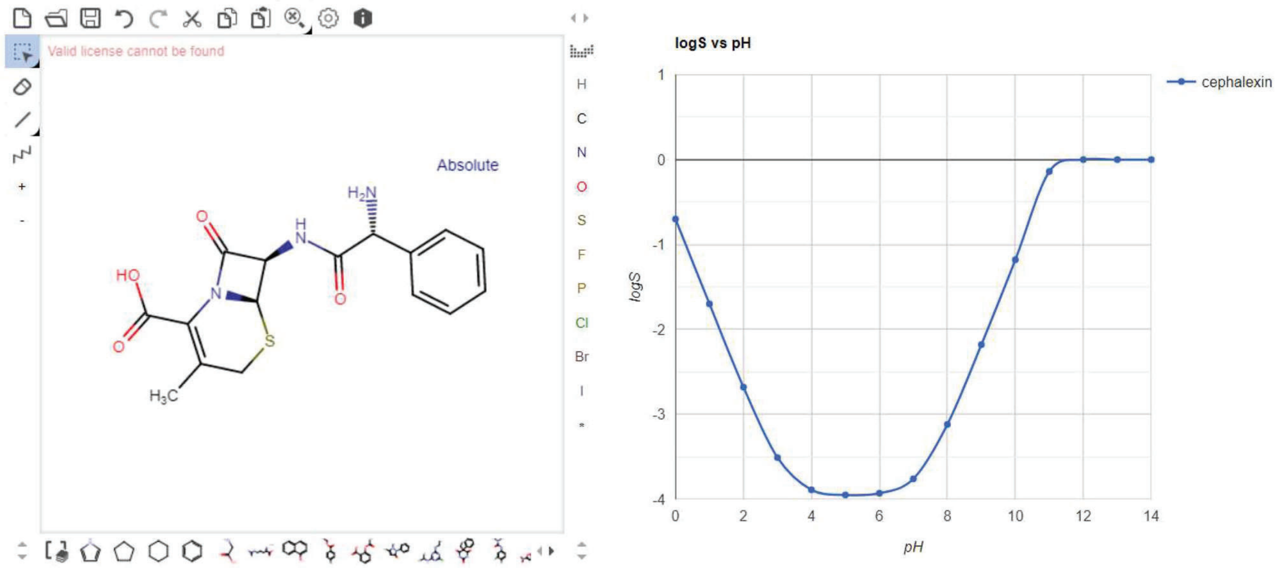

Edited by **Jitendra Belani (https://jefferson.instructure.com/courses/38086/users/233)** on Jan 13 at 1:58am
